# Genetic diversity and domestication origin of tea plant *Camellia taliensis* (Theaceae) as revealed by microsatellite markers

**DOI:** 10.1186/1471-2229-14-14

**Published:** 2014-01-09

**Authors:** Dong-wei Zhao, Jun-bo Yang, Shi-xiong Yang, Kenji Kato, Jian-ping Luo

**Affiliations:** 1 Key Laboratory for Plant Diversity and Biogeography of East Asia, Kunming Institute of Botany, Chinese Academy of Sciences, Kunming, Yunnan 650201, China; 2 University of Chinese Academy of Sciences, Beijing 100049, China; 3 Germplasm Bank of Wild Species, Kunming Institute of Botany, Chinese Academy of Sciences, Kunming, Yunnan 650201, China; 4 Graduate School of Environmental and Life Science, Okayama University, 1-1-1 Tsushima-Naka, Okayama 700-8530, Japan; 5 School of Biotechnology and Food Engineering, Hefei University of Technology, Hefei, Anhui 230009, China

**Keywords:** *Camellia taliensis*, Domestication, Genetic diversity, Microsatellite, Tea plant

## Abstract

**Background:**

Tea is one of the most popular beverages in the world. Many species in the *Thea* section of the *Camellia* genus can be processed for drinking and have been domesticated. However, few investigations have focused on the genetic consequence of domestication and geographic origin of landraces on tea plants using credible wild and planted populations of a single species. Here, *C. taliensis* provides us with a unique opportunity to explore these issues.

**Results:**

Fourteen nuclear microsatellite loci were employed to determine the genetic diversity and domestication origin of *C. taliensis*, which were represented by 587 individuals from 25 wild, planted and recently domesticated populations. *C. taliensis* showed a moderate high level of overall genetic diversity. The greater reduction of genetic diversity and stronger genetic drift were detected in the wild group than in the recently domesticated group, indicating the loss of genetic diversity of wild populations due to overexploitation and habitat fragmentation. Instead of the endangered wild trees, recently domesticated individuals were used to compare with the planted trees for detecting the genetic consequence of domestication. A little and non-significant reduction in genetic diversity was found during domestication. The long life cycle, selection for leaf traits and gene flow between populations will delay the emergence of bottleneck in planted trees. Both phylogenetic and assignment analyses suggested that planted trees may have been domesticated from the adjacent central forest of western Yunnan and dispersed artificially to distant places.

**Conclusions:**

This study contributes to the knowledge about levels and distribution of genetic diversity of *C. taliensis* and provides new insights into genetic consequence of domestication and geographic origin of planted trees of this species. As an endemic tea source plant, wild, planted and recently domesticated *C. taliensis* trees should all be protected for their unique genetic characteristics, which are valuable for tea breeding.

## Background

Plant domestication is one of the most important events in human history. People still depend on the staple cereal crops that were domesticated more than 6000 years ago in Central America [1], the Near East [2,3] and Eastern Asia [4]. In the initial domestication, the cultivated traits and genetic bottleneck may emerge in cultivars after over 1000 generations [3,5]. Reductions in genetic diversity have been found in cultivated rice [6,7], maize [8], soyabean [9,10] and other crops [11,12]. However, several instances in which no decline in the genetic diversity of planted populations occurred serve as a reminder of how complicated the situation is [13]. The biological nature of the plant whether it is annual or perennial, along with clonal propagation or sexual breeding, all have an effect on the results of domestication [14]. Differences in domestication activities, such as single or multiple domestication, also cause differences in the levels of genetic diversity in cultivars [15,16].

Both vital food plants and species that can be used for medicine or beverages, such as tea, have been domesticated for convenience. Tea was used at least as far back as 2,000 years ago in China [17]. It is one of the most popular beverages and has generated health, wealth and job opportunities throughout the world [18-20]. There are approximately 120 species in the genus *Camellia*[21], but tea in its commercial beverage form is usually produced from *C. sinensis* (L.) O. Kuntze. Since about 400 years ago when the first tea was introduced into Europe [18], *C. sinensis* has been gradually familiar to worldwide people. However, *C. taliensis* (W. W. Smith) Melchior, an important plant for use in producing tea, has only been recognized outside of its native areas for a few decades [22,23]. Several studies have investigated the genetic diversity of wild and planted trees of *C. taliensis*[24-26], and some have detected a reduction in chloroplast DNA (cpDNA) diversity during domestication [25,26]. But none of these studies has given more details on the domestication origin of this plant. Though *C. sinensis* is cultivated worldwide, there has been almost no genetic research conducted to answer the domestication questions of it, because the credible wild population of *C. sinensis* has been seldom found [27]. However, *C. taliensis* provides a unique opportunity to document the domestication origin of tea plants.

**Figure 1:**
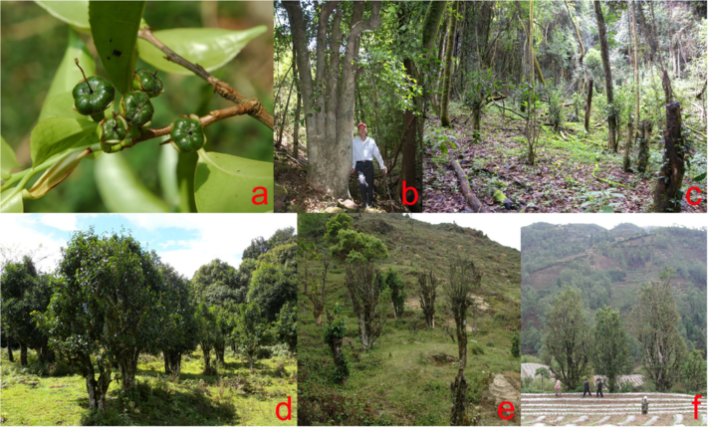
***Camellia taliensis. *(a)** A branch with young fruit showing the five-loculed ovary, **(b)** wild tree, **(c)** wild trees after felling, **(d)***in situ* recently domesticated trees, **(e)***ex situ* recently domesticated trees, **(f)** planted trees. Picture **(c)** was taken by DWZ; all other pictures were taken by SXY.

*C. taliensis*, a shrub or small tree (2–10 m) native to the subtropical mountain evergreen forests at altitudes of 1300–2700 m, is endemic from western Yunnan province of China to northern Myanmar [21]. It is generally distinguishable from *C. sinensis* by its glabrous or sparsely pubescent terminal buds and five-loculed ovary (Figure 1a). *C. sinensis* has silvery-grey sericeous terminal buds and a three-loculed ovary [21]. In western Yunnan where it is mainly found, *C. taliensis* is called ‘ye cha’ (wild tea) or ‘ben shan cha’ (local mountain tea) by the local people (Figure 1b and c) [26]. Its leaf has been collected to produce beverage that is alike the tea from *C. sinensis* var. *assamica* but has its specific characteristic constituents [28,29]. The tea probably made from *C. taliensis* was recorded 1300 years ago [17,30], and this species has been cultivated throughout western Yunnan at least for hundreds years [30]. However, many tea gardens of *C. taliensis* have been replaced by the gardens of *C. sinensis* var. *assamica* or disappeared, and the current cultivated plants of *C. taliensis* are mainly located in the Lancang River basin and Dali city [25,30].

About a dozen years ago, due to the high price that ‘wild tea’ commanded in the local market, a large number of *C. taliensis* trees in the natural forest were cut down to collect leaves [24,26]. And the phenomena of directly domesticating wild *C. taliensis* trees by clearing out the other plants on a parcel of natural forest and keeping only specimens of *C. taliensis* (Figure 1d) or digging out the wild trees and planting them in gardens (Figure 1e) had been found locally. We call these directly domesticated trees as ‘recently domesticated’. The cultivated trees derived from the seeds that gathered in tea gardens are called ‘planted’ (Figure 1f) and trees lived in the natural forest are called ‘wild’ (Figure 1b and c). Unlike the cultivated traits such as non-shattering spikelet in rice [4], tea plants do not have the clear morphological characters that may differentiate cultivated from wild trees. Rigorous field investigations and local social surveys are implemented to differentiate between cultivated and wild form of tea plants.

The archaeological evidence is usually crucial to document the domestication origins [4,31]. However, the archaeological findings associated with crop origins are limited. Widely used molecular genetics approaches such as microsatellites can now be used to determine domestication origins [32] to produce a more detailed crop history [16]. It will provide an accurate outline of the domestication process when genetic analyses are consilient with ethnobotanical approaches in the research [14]. In the present study, we provide the analyses of genetic diversity and population structure in the wild, planted and recently domesticated populations of *C. taliensis* based on 14 nuclear microsatellite makers. We aimed to assess the relative levels of genetic diversity of *C. taliensis* compared to that of *C. sinensis*, which have been investigated using landraces and improved cultivars [33,34]. Then, we discussed whether reduction of genetic diversity occurred in the planted populations of *C. taliensis* relative to the wild populations, and estimated the genetic consequence of domestication. Finally, we addressed the geographical origin of planted trees, and tried to discuss more details about the domestication process. As an endemic tea source plant, knowledge of population genetics and domestication history of *C. taliensis* is of great importance for the effective conservation and utilization of the landraces and wild germplasm and to facilitate the genetic improvement of tea plants.

## Results

### Genetic diversity and variance

A total of 178 alleles were detected in 25 populations of *C. taliensis* for the 14 loci analysed (Additional file 1). The average number of alleles per locus was 12.7. There were 15 private alleles in nine populations, including 12 alleles in the wild group and three alleles in the recently domesticated group. There were no private alleles in the planted group (Table 1), suggesting a common gene pool shared by planted and natural trees. The rare alleles (frequency ≤ 0.05) [35] accounted for 109 (61.2%) of the total 178 alleles revealed in all loci.

**Table 1:** **Genetic diversity, inbreeding coefficient and number of private alleles in each population of *C. taliensis***

**Group**	**Code**	***n***	***A***	***H***_***S***_	***H***_***O***_	***F***_***is***_	***N***_***P***_
W	SJW	24	4.202 (0.545)	0.583 (0.072)	0.506 (0.062)	0.131 (0.094)	4
	YXW	23	4.923 (0.645)	0.603 (0.086)	0.504 (0.065)	0.164 (0.071)	1
	NMW	22	4.757 (0.522)	0.619 (0.061)	0.555 (0.058)	0.104 (0.120)	0
	GMW	24	4.515 (0.530)	0.598 (0.071)	0.482 (0.053)	0.195 (0.073)	1
	CYW	23	4.196 (0.617)	0.542 (0.087)	0.467 (0.063)	0.137 (0.080)	0
	MHW	22	5.400 (0.559)	0.668 (0.065)	0.600 (0.052)	0.102 (0.059)	2
	JCW	11	3.428 (0.388)	0.541 (0.059)	0.468 (0.073)	0.135 (0.103)	2
	MJW	24	4.662 (0.498)	0.632 (0.068)	0.538 (0.050)	0.149 (0.075)	0
	OJW	23	4.641 (0.577)	0.587 (0.076)	0.466 (0.057)	0.206 (0.070)	0
	LXW	32	4.592 (0.589)	0.549 (0.079)	0.476 (0.055)	0.133 (0.050)	2
	YJW	29	4.568 (0.492)	0.623 (0.069)	0.484 (0.059)	0.223 (0.060)	0
	LCW	23	4.143 (0.525)	0.561 (0.067)	0.462 (0.052)	0.176 (0.082)	0
	HQW	33	3.883 (0.482)	0.489 (0.062)	0.397 (0.040)	0.188 (0.057)	0
	TCW	24	3.994 (0.480)	0.551 (0.076)	0.400 (0.061)	0.275 (0.098)	0
	GSW	21	4.325 (0.481)	0.585 (0.073)	0.477 (0.057)	0.184 (0.078)	0
	YDW	22	4.176 (0.517)	0.633 (0.055)	0.561 (0.051)	0.113 (0.111)	0
P	YXP	21	5.443 (0.664)	0.641 (0.075)	0.622 (0.064)	0.029 (0.068)	0
	CNP	19	4.627 (0.571)	0.577 (0.083)	0.469 (0.070)	0.188 (0.096)	0
	FQP	24	5.108 (0.648)	0.616 (0.076)	0.508 (0.057)	0.175 (0.088)	0
	LLP	24	5.162 (0.663)	0.615 (0.072)	0.503 (0.058)	0.183 (0.085)	0
	DLP	23	4.215 (0.617)	0.568 (0.072)	0.494 (0.068)	0.129 (0.114)	0
D	OJD	24	4.846 (0.526)	0.621 (0.069)	0.500 (0.058)	0.194 (0.071)	1
	LXD	24	4.745 (0.498)	0.618 (0.063)	0.488 (0.053)	0.211 (0.122)	1
	YJD	24	5.524 (0.647)	0.682 (0.072)	0.602 (0.049)	0.117 (0.062)	1
	ZKD	24	4.857 (0.640)	0.619 (0.068)	0.525 (0.054)	0.153 (0.091)	0
Total		587	6.778 (0.753)	0.597 (0.062)	0.502 (0.050)	0.160 (0.050)	-

Population means are followed by standard error in parentheses. *n*, number of individuals; *A*, allelic richness; *H*_*S*_, gene diversity; *H*_*O*_, observed heterozygosity; *F*_*is*_, inbreeding coefficient; and *N*_*P*_, number of private alleles.

*C. taliensis* showed a moderate high level of overall gene diversity (*H*_S_ = 0.597) (Table 1). For each population analysed, the highest level of genetic diversity was found in the YJD population (allelic richness corrected for sample size: *A* = 5.524; *H*_S_ = 0.682), and the lowest in the JCW population (*A* = 3.428, *H*_S_ = 0.541). Inbreeding coefficient (*F*_is_) values of 25 populations ranged from 0.029 to 0.275. The global *F*_is_ was 0.160, suggesting a low inbreeding rate in the populations of *C. taliensis*.

In the group comparison tests, the wild group contained the greatest number of rare alleles across all three groups: only nine were absent from the wild group, whereas 40 were absent from the planted trees (χ^2^ = 25.30, df = 1, *P* < 0.001) and 44 were absent from the recently domesticated individuals (χ^2^ = 30.54, df = 1, *P* < 0.001). There was no significant difference in the number of rare alleles between the planted and recently domesticated groups (χ^2^ = 0.31, df = 1, *P* = 0.578) (Table 2). *A* was significantly lower (*P*_one tailed_ < 0.05, 5000 permutations) in the wild group (4.400) than in the planted (4.911) or recently domesticated (4.993) groups. *H*_S_ was significantly higher (*P*_one tailed_ = 0.017) in the recently domesticated group (0.634) than in the wild group (0.583). The group comparison tests of *A* and *H*_S_ indicated that wild populations had a lower level of genetic diversity compared with recently domesticated and planted populations. The observed heterozygosity (*H*_O_) and *F*_is_ showed no significant differences between the three groups (*P*_one tailed_ > 0.05). The genetic differentiation (*F*_st_) was 70% higher in the wild group than in the planted or recently domesticated groups (*P*_one tailed_ ≤ 0.01) (Table 2), indicating the more genetic variation in the larger sample of wild group.

**Table 2:** Genetic structure and genetic diversity of wild (W), planted (P) and recently domesticated (D) populations

**Parameter**		**W**	**P**	**D**	***P***		
					**W vs. P**	**W vs. D**	**P vs. D**
*N*_R_ absent	All	9	40	44	<0.001**	<0.001**	0.578
	Sub	70/77	57	64	0.074	0.065	-
*A*	All	4.400 (0.115)	4.911 (0.218)	4.993 (0.179)	0.026*	0.016*	0.401
	Sub	4.624 (0.299)/4.604 (0.036)	5.302 (0.141)	5.185 (0.339)	0.099	0.126	-
*H*_S_	All	0.583 (0.011)	0.606 (0.014)	0.634 (0.016)	0.191	0.017*	0.190
	Sub	0.594 (0.009)/0.602 (0.018)	0.628 (0.013)	0.651 (0.030)	0.250	0.133	-
*H*_O_	All	0.489 (0.013)	0.522 (0.027)	0.529 (0.026)	0.133	0.110	0.431
	Sub	0.491 (0.014)/0.474 (0.009)	0.565 (0.060)	0.551 (0.051)	0.097	0.089	-
*F*_is_	All	0.161 (0.044)	0.139 (0.075)	0.166 (0.065)	0.184	0.442	0.203
	Sub	0.173 (0.067)/0.213 (0.058)	0.100 (0.071)	0.154 (0.054)	0.077	0.118	-
*F*_st_	All	0.153 (0.010)	0.092 (0.019)	0.093 (0.011)	0.003**	0.010**	0.482
	Sub	0.147 (0.027)/0.160 (0.039)	0.071 (0.020)	0.077 (0.019)	0.100	0.083	-

*N*_R_, Number of rare alleles. All, comparisons performed between wild, planted and recently domesticated groups. Sub, means the subsample comparisons: YXP and LLP versus YXW and GSW, OJW and YJW versus OJD and YJD. Parameter means are followed by standard error in parentheses. Before ‘/’, there are values of YXW and GSW; and after it, there are values of OJW and YJW. *P < 0.05, **P < 0.01.

Subsample genetic comparisons were performed between the selected adjacent populations. In terms of the number of rare alleles absent, there was no significant difference detected in YXP and LLP versus YXW and GSW (χ^2^ = 3.19, df = 1, *P* = 0.074) or in OJW and YJW versus OJD and YJD (χ^2^ = 3.39, df = 1, *P* = 0.065). No significant difference was found in the comparisons to the other genetic parameters, including *A*, *H*_*S*_, *H*_*O*_, *F*_is_ and *F*_st_ (Table 2), suggesting the similar levels of genetic diversity, inbreeding and genetic differentiation between adjacent populations.

In the Analysis of molecular variance (AMOVA), no variation was found among wild, planted and primary domesticated groups, suggesting the same genetic basis of these groups. Most of variation was detected within populations (70.6% within individuals and 16.5% among individuals within populations) and 12.9% of variation was found among populations (Table 3).

**Table 3:** **AMOVA for different regions divided in *C. taliensis***

**Source of variation**	**d. f.**	**Sum of squares**	**Variance components**	**% of variation**	**Fixation indexes**	***P***
Wild, planted and recently domesticated groups						
Among groups	2	65.2	0.00	0.0	*F*_rt_ = −0.001	0.880
Among populations within groups	22	727.2	0.63	12.9	*F*_sr_ = 0.128	0.001
Among individuals within populations	535	2704.1	0.81	16.5	*F*_is_ = 0.190	0.001
Within individuals	560	1927.5	3.44	70.6	*F*_it_ = 0.293	0.001
Total	1119	5423.9	4.87	100.0		
Geographical cluster I, II and III						
Among clusters	2	113.5	0.08	1.5	*F*_rt_ = 0.015	0.001
Among populations within clusters	22	678.8	0.58	11.8	*F*_sr_ = 0.120	0.001
Among individuals within populations	535	2704.1	0.81	16.5	*F*_is_ = 0.190	0.001
Within individuals	560	1927.5	3.44	70.2	*F*_it_ = 0.298	0.001
Total	1119	5423.9	4.90	100.0		

### Genetic drift of each population

The mean *F* values of the wild populations ranged from 0.0991 (MHW) to 0.2686 (JCW) with an average of 0.1656. The mean *F* values of the recently domesticated populations ranged from 0.0673 (YJD) to 0.1221 (LXD) with an average of 0.1072. Population YXP (0.0677) and DLP (0.1702) had the maximum and minimum mean *F* values, respectively, of the planted populations, and the average *F* value for this group was 0.1108 (Additional file 2). The genetic drift values suggested that the genetic composition of the wild populations had changed about 1.5-fold faster than that of the planted and recently domesticated populations since they diverged from the common ancestor.

### Ancestry analysis of the planted individuals

**Figure 2:**
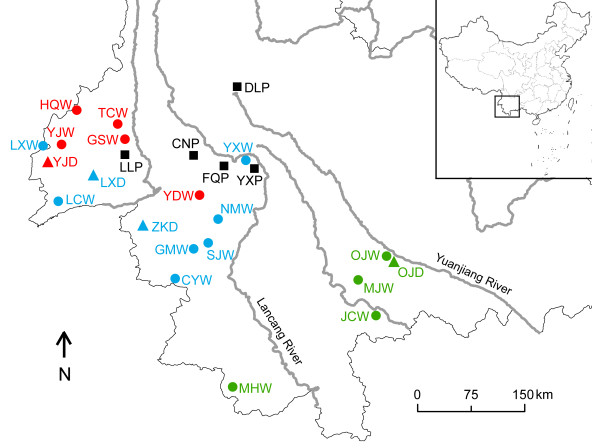
**Map of the sampling locations.** The dots indicate wild populations, the squares indicate planted populations and the triangles indicate recently domesticated populations. The colours correspond to the model ancestry analysis.

**Figure 3:**
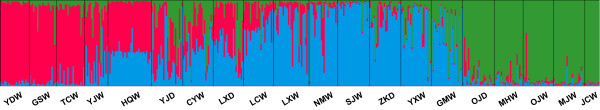
**Estimated population structure of the wild and recently domesticated *C. taliensis *with *K*** **= 3.** The genome of each individual is represented by a vertical line that is divided into coloured segments in proportion to the estimated membership of each of three clusters: Cluster I (green), Cluster II (red) and Cluster III (blue).

**Figure 4:**
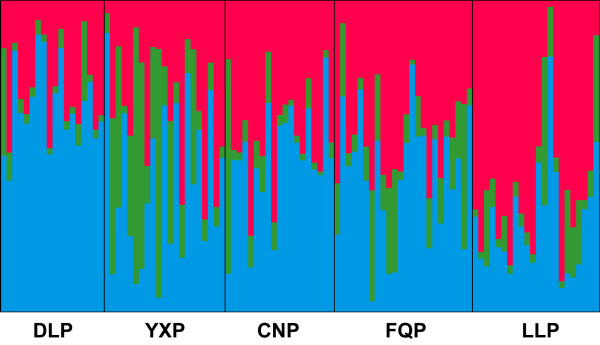
**Ancestry analysis of planted *C. taliensis.*** Each genome of a planted individual is represented by a vertical line divided into coloured segments in proportion to the estimated ancestry of each source cluster.

According to the Δ*K* method [36] using STRUCTURE, the highest likelihood for *K* was 3 (Additional file 3). Three clusters were detected in the wild and recently domesticated individuals. Cluster I of five populations (OJW, OJD, MJW, JCW and MHW) was located in southern Yunnan. The other 15 populations that were located in western Yunnan and the surrounding area were assigned into two clusters: Cluster II (HQW, TCW, GSW, YJW, YJD and YDW) was located in the northwest of this area, and the other nine populations were contained in the Cluster III (Figures 2 and 3). Planted individuals with no prior information were assigned to the three clusters. The most proportion of the planted trees genomes (62.4% of DLP, 54.6% of CNP, 51.1% of FQP, 45.4% of YXP and 25.9% of LLP) were assigned to the Cluster III, the less proportion of the planted trees genomes (63.9% of LLP, 36.6% of CNP, 34.1% of FQP, 31.4% of DLP and 27.7% of YXP) were assigned to the Cluster II and the least proportion (26.9% of YXP, 14.8% of FQP, 10.2% of LLP, 8.8% of CNP and 6.2% of DLP) to Cluster I (Figure 4). It indicated that the trees of population DLP, CNP, FQP and YXP were genetically similar to the natural individuals from Cluster III and trees of population LLP were genetically similar to those from Cluster II. However, 6.2%-26.9% of the genomes of planted trees were similar to those of Cluster I. AMOVA detected 1.5% of variation among these clusters of the whole samples, suggesting a weak differentiation at such level of *C. taliensis* (Table 3).

**Figure 5:**
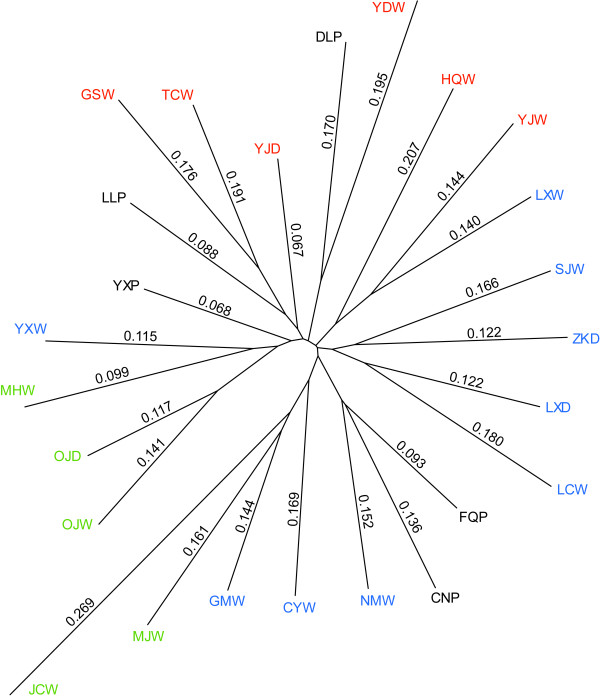
**Neighbor-joining phylogenetic relationships of 25 populations of *C. taliensis.*** Mean *F* values for each population appear along lines. The colours correspond to model source clusters.

To illustrate further the phylogenetic relationship between wild, recently domesticated and planted populations, the Neighbor-joining method was employed to reconstruct the phylogenetic tree of all 25 populations. Population FQP and CNP were phylogenetically close with population NMW, population DLP was close with population YDW, population LLP was close to population GSW and TCW, and population YXP was close to population YXW, MHW, OJD and OJW (Figure 5). Combining the phylogenetic results with genotype assignment of planted trees, it was proposed that planted trees of *C. taliensis* might have been domesticated from the central forest of western Yunnan, around the geographic area of TCW, GSW, YDW, NMW and YXW (Figure 2), and dispersed artificially to distant places.

## Discussion

### Genetic diversity of *C. taliensis*

Microsatellites had a high variation in the tea plants [33,37,38] as well as in other species of *Camellia*[39,40]. The high level of haplotype diversity and nucleotide diversity was reported by the nuclear *PAL* and cpDNA *rpl32*-*trnL* in *C. taliensis*[26]. In the present study, the overall gene diversity in *C. taliensis* (0.597) (Table 1) was lower than that reported for *C. japonica* (0.84) [39]. The gene diversity of planted *C. taliensis* (0.606) was lower than that analysed in the cultivars and six of eight landraces of *C. sinensis* in Japan (0.617-0.723) [33] but higher than that revealed in the Chinese improved cultivars of *C. sinensis* (0.588) [34]. The landraces and wild tea plants reported by Yao et al. [34] were comprised by several different species of *Camellia*, which had higher gene diversity than *C. taliensis*.

Outcrossing breeding system, long life cycle and large geographic ranges may play central roles in shaping the high genetic diversity of tea plants [41]. The lower genetic differentiation means the higher gene flow between populations [42], indicating a majority of genetic diversity preserved within populations (Table 3) [20,26]. However, human activities are the additional factors that have been impacting the genetic diversity of tea plants and the adverse effects of encroachment of humans are increasing continuously. Felling the wild trees of *C. taliensis* to collect leaves for producing the wild tea (Figure 1c) [24,26] and further deforestation to make way for farming, grazing and construction have caused persistent and serious damage to natural sources of this tea plant [43]. The lower genetic diversity and the higher *F* values in the wild populations may indicate the stronger genetic drift due to these causes (Table 2, Figure 5 and Additional file 2).

### Genetic consequence of domestication

The genetic drift analysis (Figure 5, Additional file 2) and both the group and subsample tests showed a lower level of genetic diversity in the wild populations (Table 2). Did they reveal that the planted populations have an advanced genetic diversity, which was exceptive in the plant domestication [5]? It is not possible to identify a real genetic consequence of domestication in the comparison of endangered wild populations [26] and protected planted populations. The decline in genetic diversity of wild trees introduced by human activity may mask the genetic bottleneck in planted individuals. However, the recently domesticated trees that came directly from the natural forest may partly represent the wild plants that were free from damage. Compared with the recently domesticated group, it indicates that the wild group has lost genetic diversity rather than the planted group has gained genetic diversity. Furthermore, although the differences of genetic diversity between planted and recently domesticated groups were not significant, the little reduction of *A* and *H*_*S*_ and slight growth of *F* value in planted group would indicate a little but non-significant genetic bottleneck during the domestication, which suggests the complicated situation in the tea plants domestication (Table 2, Figure 5 and Additional file 2).

The information from both the chloroplast and nuclear genomes helped us to comprehensively understand the consequence of domestication. CpDNA *rpl32-trnL* intergenic spacer analyses showed a reduction of the genetic diversity during domestication with three planted populations and 21 wild populations of *C. taliensis*[25,26]. The maternal inheritance cpDNA analysis would suggest the limited seed sources of the planted *C. taliensis* during domestication. However, the analysis of cpDNA does not always give results that are consistent with the results analysed by nuclear DNA [44]. Almost the whole cpDNA variation (98.75%) was distributed among *C. taliensis* populations, which was contrastingly different from the results detected by nuclear DNA markers (Table 3) [26]. It may be rational to consider that the sampling number of populations will affect the comparison result of cpDNA diversity between different groups [44]. The loss of cpDNA diversity in the three planted populations compared with 21 wild populations may be partly derived from the much smaller number of planted populations.

Tea plants have 5–10 years long life cycle [45], and have been selected on the traits of leaf during their domestication. The artificial selection based on leaf characteristics may have less of an impact on the genomes of tea plants, especially as they are xenogamous plants and reproduce from seed. Additionally, the gene flow among local planted, wild and recently domesticated trees would introduce introgression among different groups and reduce the genetic difference (Table 3) [42]. The planted population of DLP, which is located at the northern frontier of the natural distribution of *C. taliensis* (Figure 2), is recorded as having a long period of cultivation [45]. We did not find wild *C. taliensis* trees in the local forests in the DLP area. Isolated from natural trees and long period of cultivation seem to be the major causes of the lowest genetic diversity and the highest drift values of population DLP among the planted populations (Table 1, Figure 5 and Additional file 2). Trees in population YXP had a high genetic diversity, which may be the result of gene flow between YXP and YXW. The tea garden from which population YXP was derived contained several cultivars of *C. sinensis*. Mixed cultivation may have made genetic introgression between the two species more feasible [46].

### Geographical origins of the planted trees

In the present study, both phylogenetic and assignment analysis indicated that the planted trees of *C. taliensis* may be derived from the central forest of western Yunnan and dispersed artificially to distant places (Figures 2, 3, 4 and 5). Four of five planted populations (LLP, CNP, FQP and YXP) came from this area, suggesting that *C. taliensis* has been mainly domesticated from the adjacent natural forests. This area has a long period of domesticating tea plants [30]. A legend of ‘dou cha’ (tea fight) could be heard in the village of population YXP. Taking with the tea and seeds, people came from different places gathered in the village for the tea fight. The person who won the competition had to supply their elite seeds of *C. taliensis* to others for planting [30]. Through these human activities, the landraces of *C. taliensis* had been selected and spread to the farther place. The artificial dispersal of landraces would explain the close genetic relationship between some planted trees in population YXP and the natural trees of Cluster I in southern Yunnan as well as the relationship between DLP and YDW (Figures 2, 4 and 5).

Most of the crops that spread worldwide due to their unique values were initially derived from the native habitats of their wild ancestors, which can be traced back through both archaeological and genetic approaches [16,31]. The *in situ* plant domestication process is still underway [47]. From the current activities of recent domestication, it may be reasonable to consider that the origin of planted trees of *C. taliensis* was not a single event but an extended multistage process in which wild trees added sequentially over hundreds of years. The non-significant reduction in genetic diversity of planted trees will support this inference (Table 2). However, in the field investigation and local social survey, we found that a large number of endemic planted trees of *C. taliensis* had been replaced by the ecdemic improved cultivars of *C. sinensis* var. *assamica* in the late one hundred years [25]. It suggested that improved cultivars of tea were valued for their higher quality. In the last dozen years, the domestication of wild *C. taliensis* was principally owing to the high price of wild tea that had been hyped with the cultural values and it was claimed to be produced without using pesticide and chemical fertilizers [24,26]. It is hard to believe that people were willing to abandon the improved landrace in their gardens but introduce the wild trees from the natural forests frequently during hundreds of years. It is considered that the more likely process is the successive domestication in tea gardens and accompanied occasional introduction of few wild seeds or seedlings of *C. taliensis*.

### Conservation strategies and utilization in tea breeding

Although the planted and recently domesticated populations had a greater genetic diversity, it is the wild populations that have preserved the most private alleles and rare alleles, making them the most important reservoirs of genetic variation (Tables 1 and 2). Taking natural trees and planting them in private gardens or clearing out other shrubs and transforming a plot of wild forest into one’s own tea garden destroys not only wild resources of *C. taliensis*, but also the natural forest in general (Figure 1d and e). Without effective restriction, each individual action of initial domestication would add up to the substantial damage of common resources, not unlike the tragedy of the commons described by Hardin [48].

It is essential to conserve common natural resources, including wild tea plants, through efficient management. However, *C. taliensis* is an important landrace source that could generate new developments in tea breeding, for which wild genetic resources should be indispensable. The paradox of protection and production could be addressed through rapid reproduction from cuttings [49] of wild trees. The planted trees of *C. taliensis* should also be protected for their selected genetic characteristics and endemic culture, and they will facilitate the further breeding of tea plants.

## Conclusions

In this study, we firstly illustrated the domestication origin of a tea plant with genetic approaches. Fourteen nuclear microsatellite loci detected a moderate high genetic diversity in *C. taliensis*. Using the credible wild, planted and recently domesticated populations of this tea plant, we discussed the genetic consequence of domestication and geographic origin of the planted trees. Group and subsample tests indicated that a little and non-significant bottleneck occurred during the domestication. The phylogenetic and assignment analyses suggested that the planted trees may have been domesticated from the adjacent central forest of western Yunnan and dispersed artificially to distant places. As an important tea source plant in Yunnan province of China, *C. taliensis* should be protected and utilized for their unique genetic characteristics, which are valuable for the genetic improvement of tea plants. Our study will be helpful to distinguish the genetic results of different collection and domestication activities of tea plant, and will further give deep insights into the custom and history of tea domestication.

## Methods

### Sampling of *C. taliensis*

Our sampling localities encompassed almost the entire range of *C. taliensis* in western Yunnan and the surrounding areas (Figure 2, Additional file 4). Wild trees were sampled from the natural forests (Figure 1b and c). Planted trees were collected from tea gardens and identified as seedling plants (Figure 1f). Recently domesticated trees were sampled in tea gardens and the owners verified them as having come directly from natural forests (Figure 1d and e). We collected 587 individual plants from 16 wild populations (W), five planted populations (P) and four recently domesticated populations (D). Leaves were preserved in silica gel for DNA extraction. Voucher specimens were deposited at the Herbarium of the Kunming Institute of Botany, Chinese Academy of Sciences (KUN) (Additional file 4).

### DNA extraction and microsatellite analysis

Total genomic DNA was extracted using a modified protocol of Doyle and Doyle [50]. Thirty-seven primer sets were selected from the known microsatellite loci in *C. taliensis*[51], *C. sinensis*[37,52,53], *C. japonica*[54] and other species [55]. After the primary screening, we got 14 nuclear microsatellite loci in which only five primer sets were transferred from other species of *Camellia* as rest of the primer sets were developed in specific for *C. taliensis* (Additional file 1). In the selected 37 primer sets, there were two chloroplast microsatellite loci: ccmp6 [52] and PS-ID [55]. We had not found the mutation in PS-ID from primary screening, but had found one mutation in ccmp6. However, when we developed all 587 individual with ccmp6, there was only a single mutation in population TCW. So, these two chloroplast microsatellite loci had not been implemented in the subsequent analyses.

PCR amplification was carried out according to the standard protocol and the products were separated on 8% polyacrylamide denaturing gel by silver staining. Two or three samples of each primer set were sequenced to ensure the markers hitting the same microsatellites regions as reported. The alleles were scored according to the specific references that contained 1–5 alleles of each locus from single or mixed PCR products and the100 bp DNA ladder (Tiangen Biotech, Beijing, China). About 30% of total data was performed additionally on the gel for cross and repeated scoring.

### Genetic diversity estimation

The differences between the number of rare alleles (frequency ≤ 0.05) [35] present in the wild populations, planted populations and recently domesticated populations were tested using a χ^2^ contingency table test [56]. The allelic richness corrected for sample size (*A*), the observed heterozygosity (*H*_O_), the gene diversity (*H*_S_) and the *F*-statistics were determined in FSTAT V2.9.3.2 [57]. This program was also used to perform comparison tests between each genetic parameter of the wild, planted and recently domesticated groups. The one-tailed *P* values were estimated using the random permutation method.

In the group comparison tests, the wide differences in number of trees between wild and planted groups as well as between wild and recently domesticated groups may bias the results. The subsample tests had been developed for avoiding this potential statistic bias and achieving the more detailed results. Certain adjacent populations in different groups were selected to perform the subsample comparisons: YXP and LLP versus YXW and GSW, OJW and YJW versus OJD and YJD (Figure 2). These genetic comparisons were also carried out in FSTAT V2.9.3.2 [57].

### Genetic drift analysis

The *F* model, performed with the program STRUCTURE V2.3.3 [58], was used to estimate the rate of drift away from a common ancestor for each wild, planted and recently domesticated populations. A Bayesian approach was implemented to infer the ancestral allele frequencies and the rates of drift away from the ancestral allelic state in each population (*F* values). For all pairs of wild, planted and recently domesticated populations, we set the prior mean *F* to 0.1. Three parallel Markov chains were run with a burn-in of 10^4^ iterations and a run length of 10^5^ iterations for each comparison. Regions of 90% credibility were computed from the distribution of *F* values estimated in the final run. The mean *F* values for each population were calculated across all runs and all other populations that belonged to different groups [32].

### Ancestry analysis of the planted trees

Using the program STRUCTURE V2.3.3 [58,59], we estimated the number of genetic clusters of natural *C. taliensis* to which we would assign the planted trees. Both the wild and recently domesticated samples were included to estimate the genetic clusters, because the recently domesticated trees came directly from the natural forest and the broader natural samples would make the subsequence assignment analysis more accurate. We used the admixture model and assumed that the allele frequencies were correlated among the populations. The simulations were run with a burn-in of 500,000 iterations and a run length of 10^6^ iterations from *K* = 1 through 20. Runs for each *K* were replicated 10 times and the true *K* was determined according to the method described by Evanno et al. [36]. After deduction of true *K* value, the wild and recently domesticated individuals were specified as belonging to each of *K* clusters but no prior information was specified as to the origin of planted trees, which established a new dataset. Using this new dataset and the admixture model, ten parallel Markov chains were run for the correlated allele frequency models with a burn-in of 500,000 iterations and a run length of 10^6^ iterations to estimate the proportion of every planted tree’s genome possessing ancestry in each of *K* clusters [10,32]. The results of the genetic clustering and ancestry analysis were perfected in the programs CLUMPP V1.1.2 [60] and DISTRUCT [61].

### Phylogenetic analysis

Genetic distances (*D*_A_) [62] between all 25 populations were calculated by DISPAN [63] with 1000 replicate bootstrap data sets. Using the pairwise *D*_A_, the program MEGA 5.1 [64] was implemented to construct a Neighbor-joining tree of the 25 populations.

### Analysis of molecular variance

Analysis of molecular variance (AMOVA) was performed with GENALEX 6.501 [65,66] to detect the proportion of variance of wild, planted and recently domesticated groups. After each planted population was assigned to the geographic cluster, GENALEX 6.501 was also implemented to analyse the proportion of genetic variance in these clusters.

## Competing interests

The authors declare that they have no competing interests.

## Authors’ contributions

DWZ participated in the field sample collection, carried out the molecular genetic studies, performed the statistical analysis and drafted the manuscript. JBY participated in the molecular genetic studies and statistical analysis. SXY designed the research, collected samples, participated in the molecular genetic studies and revised the manuscript. KK involved the molecular genetic studies and revised the manuscript. JPL involved the molecular genetic studies. All authors read and approved the final manuscript.

## Supplementary Material

Additional file 1 Description of the microsatellite loci. Click here for file

Additional file 2 **Results of the *F* model comparisons.** F1, F2 and F3 refer to the *F* values of the wild, planted and recently domesticated populations, respectively. Click here for file

Additional file 3 **Results of detecting the true *K.***
Click here for file

Additional file 4 **Sampling localities of *Camellia taliensis.*** Population LXW is located in Myanmar and the other populations are located in Yunnan province of China. Click here for file

## References

[B1] Ranere AJ Piperno DR Holst I Dickau R Iriarte J The cultural and chronological context of early Holocene maize and squash domestication in the Central Balsas River Valley, Mexico Proc Natl Acad Sci USA 2009 106 5014 5018 10.1073/pnas.0812590106 19307573 PMC2664064

[B2] Heun M Schäfer-Pregl R Klawan D Castagna R Accerbi M Borghi B Salamini F Site of einkorn wheat domestication identified by DNA fingerprinting Science 1997 278 1312 1314 10.1126/science.278.5341.1312

[B3] Tanno KI Willcox G How fast was wild wheat domesticated? Science 1886 2006 311 10.1126/science.1124635 16574859

[B4] Fuller DQ Qin L Zheng YF Zhao ZJ Chen XG Hosoya LA Sun GP The domestication process and domestication rate in rice: spikelet bases from the Lower Yangtze Science 2009 323 1607 1610 10.1126/science.1166605 19299619

[B5] Doebley JF Gaut BS Smith BD The molecular genetics of crop domestication Cell 2006 127 1309 1321 10.1016/j.cell.2006.12.006 17190597

[B6] Zhu QH Zheng XM Luo JC Gaut BS Ge S Multilocus analysis of nucleotide variation of *Oryza sativa* and its wild relatives: severe bottleneck during domestication of rice Mol Biol Evol 2007 24 875 888 17218640 10.1093/molbev/msm005

[B7] Li ZM Zheng XM Ge S Genetic diversity and domestication history of African rice (*Oryza glaberrima*) as inferred from multiple gene sequences Theor Appl Genet 2011 123 21 31 10.1007/s00122-011-1563-2 21400109

[B8] Tenaillon MI U’Ren J Tenaillon O Gaut BS Selection versus demography: a multilocus investigation of the domestication process in maize Mol Biol Evol 2004 21 1214 1225 10.1093/molbev/msh102 15014173

[B9] Hyten DL Song QJ Zhu YL Choi IY Nelson RL Costa JM Specht JE Shoemaker RC Cregan PB Impacts of genetic bottlenecks on soybean genome diversity Proc Natl Acad Sci USA 2006 103 16666 16671 10.1073/pnas.0604379103 17068128 PMC1624862

[B10] Guo J Wang YS Song C Zhou JF Qiu LJ Huang HW Wang Y A single origin and moderate bottleneck during domestication of soybean (*Glycine max*): implications from microsatellites and nucleotide sequences Ann Bot 2010 106 505 514 10.1093/aob/mcq125 20566681 PMC2924825

[B11] Bourguiba H Audergon JM Krichen L Trifi-Farah N Mamouni A Trabelsi S D’Onofrio C Asma BM Santoni S Khadari B Loss of genetic diversity as a signature of apricot domestication and diffusion into the Mediterranean Basin BMC Plant Biol 2012 12 49 10.1186/1471-2229-12-49 22510209 PMC3511222

[B12] Chapman MA Burke JM **DNA sequence diversity and the origin of cultivated safflower (**** *Carthamus tinctorius * ****L.; Asteraceae)** BMC Plant Biol 2007 7 60 10.1186/1471-2229-7-60 17986334 PMC2241619

[B13] Kelly BA Hardy OJ Bouvet JM Temporal and spatial genetic structure in *Vitellaria paradoxa* (shea tree) in an agroforestry system in southern Mali Mol Ecol 2004 13 1231 1240 10.1111/j.1365-294X.2004.02144.x 15078458

[B14] Parra F Casas A Peñaloza-Ramírez JM Cortés-Palomec AC Rocha-Ramírez V González-Rodríguez A Evolution under domestication: ongoing artificial selection and divergence of wild and managed *Stenocereus pruinosus* (Cactaceae) populations in the Tehuacán Valley, Mexico Ann Bot 2010 106 483 496 10.1093/aob/mcq143 20729372 PMC2924835

[B15] Miller A Schaal B Domestication of a Mesoamerican cultivated fruit tree, *Spondias purpurea* Proc Natl Acad Sci USA 2005 102 12801 12806 10.1073/pnas.0505447102 16126899 PMC1200284

[B16] Kilian B Özkan H Walther A Kohl J Dagan T Salamini F Martin W Molecular diversity at 18 loci in 321 wild and 92 domesticate lines reveal no reduction of nucleotide diversity during *Triticum monococcum* (einkorn) domestication: implications for the origin of agriculture Mol Biol Evol 2007 24 2657 2668 10.1093/molbev/msm192 17898361

[B17] Fang J No tea before the Warring States period Agricultural History of China 1998 17 6 14 39

[B18] Jackson JR Tea Nature 1870 2 215 217 10.1038/002215a0

[B19] Jankun J Selman SH Swiercz R Skrzypczak-Jankun E Why drinking green tea could prevent cancer Nature 1997 387 561 10.1038/42381 9177339

[B20] Paul S Wachira FN Powell W Waugh R Diversity and genetic differentiation among populations of Indian and Kenyan tea [*Camellia sinensis* (L.) O. Kuntze] revealed by AFLP markers Theor Appl Genet 1997 94 255 263 10.1007/s001220050408

[B21] Min TL Bartholomew B Wu ZY, Raven PH, Hong DY Theaceae Flora of China. Volume 12 2007 Beijing and St. Louis: Science Press and Missouri Botanical Garden 367 412

[B22] Kingdon-Ward F Does wild tea exist? Nature 1950 165 297 299

[B23] Wight W Barua PK What is Tea? Nature 1957 179 506 507 10.1038/179506a0

[B24] Ji PZ Wang YG Jiang HB Tang YC Wang PS Zhang J Huang XQ Genetic diversity of *Camellia taliensis* from Yunnan province of China revealed by AFLP analysis J Tea Sci 2009 29 329 335

[B25] Liu Y Yang SX Gao LZ Comparative study on the chloroplast *RPL32-TRNL* nucleotide variation within and genetic differentiation among ancient tea plantations of *Camellia sinensis* var. *assamica* and *C. taliensis* (Theaceae) from Yunnan, China Acta Botanica Yunnanica 2010 32 427 434

[B26] Liu Y Yang SX Ji PZ Gao LZ Phylogeography of *Camellia taliensis* (Theaceae) inferred from chloroplast and nuclear DNA: insights into evolutionary history and conservation BMC Evol Biol 2012 12 92 10.1186/1471-2148-12-92 22716114 PMC3495649

[B27] Chen J Pei SJ Studies on the origin of tea cultivation Acta Botanica Yunnanica 2003 Suppl XIV 33 40

[B28] Gao DF Zhang YJ Yang CR Chen KK Jiang HJ Phenolic antioxidants from green tea produced from *Camellia taliensis* J Agric Food Chem 2008 56 7517 7521 10.1021/jf800878m 18636681

[B29] Zhu LF Dong HZ Yang SX Zhu HT Xu M Zeng SF Yang CR Zhang YJ Chemical compositions and antioxidant activity of essential oil from green tea produced from *Camellia taliensis* (Theaceae) in Yuanjiang, Southwestern China Plant Divers Res 2012 34 409 416

[B30] Yang CR Zhang YJ Gao DF Chen KK Jiang HJ Assessment of germplasm of *Camellia taliensis* and origin of cultivated *C. sinensis* var. *assamica* Tea Sci Technol 2008 3 1 4

[B31] Benz BF Archaeological evidence of teosinte domestication from Guilá Naquitz, Oaxaca Proc Natl Acad Sci USA 2001 98 2104 2106 10.1073/pnas.98.4.2104 11172083 PMC29389

[B32] Harter AV Gardner KA Falush D Lentz DL Bye RA Rieseberg LH Origin of extant domesticated sunflowers in eastern North America Nature 2004 430 201 205 10.1038/nature02710 15241413

[B33] Ohsako T Ohgushi T Motosugi H Oka K Microsatellite variability within and among local landrace populations of tea, *Camellia sinensis* (L.) O. Kuntze, in Kyoto, Japan Genet Resour Crop Evol 2008 55 1047 1053 10.1007/s10722-008-9311-4

[B34] Yao MZ Ma CL Qiao TT Jin JQ Chen L Diversity distribution and population structure of tea germplasms in China revealed by EST-SSR markers Tree Genet Genomes 2012 8 205 220 10.1007/s11295-011-0433-z

[B35] White GM Boshier DH Powell W Genetic variation within a fragmented population of *Swietenia humilis* Zucc Mol Ecol 1999 8 1899 1909 10.1046/j.1365-294x.1999.00790.x 10620233

[B36] Evanno G Regnaut S Goudet J Detecting the number of clusters of individuals using the software STRUCTURE: a simulation study Mol Ecol 2005 14 2611 2620 10.1111/j.1365-294X.2005.02553.x 15969739

[B37] Freeman S West J James C Lea V Mayes S Isolation and characterization of highly polymorphic microsatellites in tea (*Camellia sinensis*) Mol Ecol Notes 2004 4 324 326 10.1111/j.1471-8286.2004.00682.x

[B38] Sharma RK Bhardwaj P Negi R Mohapatra T Ahuja PS **Identification, characterization and utilization of unigene derived microsatellite markers in tea (**** *Camellia sinensis * ****L.)** BMC Plant Biol 2009 9 53 10.1186/1471-2229-9-53 19426565 PMC2693106

[B39] Ueno S Tomaru N Yoshimaru H Manabe T Yamamoto S Genetic structure of *Camellia japonica* L. in an old-growth evergreen forest, Tsushima, Japan Mol Ecol 2000 9 647 656 10.1046/j.1365-294x.2000.00891.x 10849281

[B40] Wei JQ Chen ZY Wang ZF Tang H Jiang YS Wei X Li XY Qi XX Isolation and characterization of polymorphic microsatellite loci in *Camellia nitidissima* Chi (Theaceae) Am J Bot 2010 97 e89 e90 10.3732/ajb.1000234 21616788

[B41] Hamrick JL Godt MJW Sherman-Broyles SL Factors influencing levels of genetic diversity in woody plant species New For 1992 6 95 124 10.1007/BF00120641

[B42] Hübner S Günther T Flavell A Fridman E Graner A Korol A Schmid KJ Islands and streams: clusters and gene flow in wild barley populations from the Levant Mol Ecol 2012 21 1115 1129 10.1111/j.1365-294X.2011.05434.x 22256891

[B43] Jiang HB Wang YG Tang YC Song WX Li YY Ji PZ Huang XQ Investigation of wild tea plant (*Camellia taliensis*) germplasm resource from Yunnan, China Southwest China J Agri Sci 2009 22 1153 1157

[B44] Petit RJ Duminil J Fineschi S Hampe A Salvini D Vendramin GG Comparative organization of chloroplast, mitochondrial and nuclear diversity in plant populations Mol Ecol 2005 14 689 701 15723661 10.1111/j.1365-294X.2004.02410.x

[B45] Min TL Monograph of the Genus Camellia 2000 Kunming: Yunnan Science and Technology Press

[B46] Zhao DW Yang SX Rediscovery of *Camellia grandibracteata* (Theaceae) with emendate description J Trop Subtrop Bot 2012 20 399 402

[B47] Casas A Otero-Arnaiz A Pérez-Negrón E Valiente-Banuet A *In situ* management and domestication of plants in Mesoamerica Ann Bot 2007 100 1101 1115 10.1093/aob/mcm126 17652338 PMC2759202

[B48] Hardin G The tragedy of the commons Science 1968 162 1243 1248 5699198

[B49] Qin XJ Lin CC Chen XQ Studies on new techniques of cuttage reproduction and coming out from nursery rapidly of tea Chin Agri Sci Bull 2004 20 224 226

[B50] Doyle JJ Doyle JL A rapid DNA isolation procedure for small quantities of fresh leaf tissue Phytochem Bull 1987 19 11 15

[B51] Yang JB Yang J Li HT Zhao Y Yang SX Isolation and characterization of 15 microsatellite markers from wild tea plant (*Camellia taliensis*) using FIASCO method Conserv Genet 2009 10 1621 1623 10.1007/s10592-009-9814-3

[B52] Kaundun SS Matsumoto S Heterologous nuclear and chloroplast microsatellite amplification and variation in tea, *Camellia sinensis* Genome 2002 45 1041 1048 10.1139/g02-070 12502248

[B53] Hung CY Wang KH Huang CC Gong X Ge XJ Chiang TY Isolation and characterization of 11 microsatellite loci from *Camellia sinensis* in Taiwan using PCR-based isolation of microsatellite arrays (PIMA) Conserv Genet 2008 9 779 781 10.1007/s10592-007-9391-2

[B54] Ueno S Yoshimaru H Tomaru N Yamamoto S Development and characterization of microsatellite markers in *Camellia japonica* L Mol Ecol 1999 8 335 346 10065549 10.1046/j.1365-294x.1999.00534.x

[B55] Nakamura I Urairong H Kameya N Fukuta Y Chitrakon S Sato YI Six different plastid subtypes were found in *O. sativa*–*O. rufipogon* complex Rice Gen Newslett 1998 15 80 82

[B56] Jump AS Peñuelas J Genetic effects of chronic habitat fragmentation in a wind-pollinated tree Proc Natl Acad Sci USA 2006 103 8069 8100 10.1073/pnas.0510127103 PMC1472435 16698935

[B57] Goudet J FSTAT (version 1.2): a computer program to calculate F-statistics J Hered 1995 86 485 486

[B58] Falush D Stephens M Pritchard JK Inference of population structure using multilocus genotype data: linked loci and correlated allele frequencies Genetics 2003 164 1567 1587 12930761 10.1093/genetics/164.4.1567 PMC1462648

[B59] Pritchard JK Stephens M Donnelly P Inference of population structure using multilocus genotype data Genetics 2000 155 945 959 10835412 10.1093/genetics/155.2.945 PMC1461096

[B60] Jakobsson M Rosenberg NA CLUMPP: a cluster matching and permutation program for dealing with label switching and multimodality in analysis of population structure Bioinformatics 2007 23 1801 1806 10.1093/bioinformatics/btm233 17485429

[B61] Rosenberg NA DISTRUCT: a program for the graphical display of population structure Mol Ecol Notes 2004 4 137 138

[B62] Nei M Tajima F Tateno Y Accuracy of estimated phylogenetic trees from molecular data J Mol Evol 1983 19 153 170 10.1007/BF02300753 6571220

[B63] Ota T DISPAN: Genetic distance and phylogenetic analysis [http://www.softpedia.com/get/Science-CAD/DISPAN.shtml]

[B64] Tamura K Peterson D Peterson N Stecher G Nei M Kumar S MEGA5: molecular evolutionary genetics analysis using maximum likelihood, evolutionary distance, and maximum parsimony methods Mol Biol Evol 2011 28 2731 2739 10.1093/molbev/msr121 21546353 PMC3203626

[B65] Peakall R Smouse PE GENALEX 6: genetic analysis in Excel. Population genetic software for teaching and research Mol Ecol Notes 2006 6 288 295 10.1111/j.1471-8286.2005.01155.x PMC3463245 22820204

[B66] Peakall R Smouse PE GenAlEx 6.5: genetic analysis in Excel. Population genetic software for teaching and research—an update Bioinformatics 2012 28 2537 2539 10.1093/bioinformatics/bts460 22820204 PMC3463245

