# Supplementary material for: Genetic diversity and domestication origin of tea plant Camellia taliensis (Theaceae) as revealed by microsatellite markers
Source: BMC Plant Biol. 2014 Jan 9;14:14. doi: 10.1186/1471-2229-14-14 (PMC3890520; doi:10.1186/1471-2229-14-14)
Supplement: Additional file 1 — Description of the microsatellite loci. [file 1471-2229-14-14-S1.doc]

**Additional file 1:** Description of the microsatellite loci.

| NO. | Locus | Repeat motif | Number of alleles | *Tm* (℃) | *H_S_* | *H_O_* | Source species |
| --- | --- | --- | --- | --- | --- | --- | --- |
| 1 | MSCjaF37 (M37) | (AG)_13_(GAA)_7_ | 21 | 58 | 0.826 | 0.627 | *C. japonica* [1] |
| 2 | MSCjaH38 (M38) | (GA)_14_ | 13 | 55 | 0.669 | 0.409 |
| 3 | CamsinM4 (M4) | (GA)_19_ | 12 | 52 | 0.703 | 0.504 | *C. sinensis* [2] |
| 4 | CamsinM5 (M5) | (GT)_15_(GA)_8_ | 14 | 53 | 0.562 | 0.489 |
| 5 | CamsinM11 (M11) | (CA)_12_ | 17 | 53 | 0.611 | 0.830 |
| 6 | A37 | (AGG)_9_ | 13 | 54 | 0.640 | 0.562 | *C. taliensis* [3] |
| 7 | C5 | (AC)_12_ | 6 | 55 | 0.165 | 0.209 |
| 8 | G19 | (AG)_13_ | 14 | 55 | 0.784 | 0.690 |
| 9 | G23 | (CT)_5_ | 4 | 56 | 0.254 | 0.291 |
| 10 | G47 | (AG)_5_ | 7 | 51 | 0.152 | 0.131 |
| 11 | G74 | (CT)_12_ | 13 | 55 | 0.756 | 0.582 |
| 12 | G79 | (CT)_11_ | 10 | 57 | 0.692 | 0.561 |
| 13 | Q5 | (CT)_15_ | 16 | 54 | 0.795 | 0.576 |
| 14 | Q6 | (AG)_23_ | 18 | 58 | 0.743 | 0.568 |

1. Ueno S, Yoshimaru H, Tomaru N, Yamamoto S: **Development and characterization of microsatellite markers in *Camellia japonica* L.** *Mol Ecol* 1999, **8:**335-346.

2. Freeman S, West J, James C, Lea V, Mayes S: **Isolation and characterization of highly polymorphic microsatellites in tea (*Camellia sinensis*).** *Mol Ecol Notes* 2004, **4:**324-326.

3. Yang JB, Yang J, Li HT, Zhao Y, Yang SX: **Isolation and characterization of 15 microsatellite markers from wild tea plant (*Camellia taliensis*) using FIASCO method.** *Conserv Genet* 2009, **10:**1621-1623.
