# Supplementary material for: Genetic diversity and domestication origin of tea plant Camellia taliensis (Theaceae) as revealed by microsatellite markers
Source: BMC Plant Biol. 2014 Jan 9;14:14. doi: 10.1186/1471-2229-14-14 (PMC3890520; doi:10.1186/1471-2229-14-14)
Supplement: Additional file 4 — Sampling localities of Camellia taliensis. Population LXW is located in Myanmar and the other populations are located in Yunnan province of China. [file 1471-2229-14-14-S4.doc]

**Additional file 4:** Sampling localities of *Camellia taliensi*s.

| Code | Locality | Voucher specimens | Lat. (N) | Long. (E) |
| --- | --- | --- | --- | --- |
| SJW | Mengku, Shuangjiang county | S.-X. Yang 06A10 | 23°39.2´ | 99°47.6´ |
| YXW | Manwan, Yunxian county | S.-X. Yang 06B88 | 24°37.9´ | 100°19.1´ |
| NMW | Nanmei, Lincang city | S.-X. Yang & D.-W. Zhao 07A26 | 23°54.9´ | 99°54.2´ |
| GMW | Manghong, Gengma county | S.-X. Yang & D.-W. Zhao 07A27 | 23°34.3´ | 99°38.4´ |
| CYW | Shanjia, Cangyuan county | S.-X. Yang & D.-W. Zhao 07A63 | 23°10.2´ | 99°24.0´ |
| MHW | Bada, Menghai county | S.-X. Yang & Y.-M. Wu 07B28 | 21°50.0´ | 100°6.2´ |
| JCW | Jiahe, Jiangcheng county | S.-X. Yang & Y.-M. Wu 07B33 | 22°43.6´ | 101°54.5´ |
| MJW | Yayi, Mojiang county | S.-X. Yang & Y.-M. Wu 07B53 | 23°10.5´ | 101°42.0´ |
| OJW | Yangjie, Yuanjiang county | S.-X. Yang & Y.-M. Wu 07B86 | 23°26.3´ | 102°3.5´ |
| LXW | Lexin, Kachin, Myanmar | S.-X. Yang & Y. Liu 08A07 | 24°50.2´ | 97°44.0´ |
| YJW | Mengnong, Yingjiang county | S.-X. Yang & Y. Liu 08A08 | 24°53.6´ | 97°56.2´ |
| LCW | Jinghan, Longchuan county | S.-X. Yang & Y. Liu 08A14 | 24°10.5´ | 97°56.2´ |
| HQW | Houqiao, Tengchong county | S.-X. Yang & Y. Liu 08A49 | 25°17.8´ | 98°7.3´ |
| TCW | Dahaoping, Tengchong county | S.-X. Yang & Y. Liu 08A52 | 24°56.9´ | 98°44.7´ |
| GSW | Gaoligongshan, Tengchong county | S.-X. Yang & Y. Liu 08A53 | 25°8.2´ | 98°39.0´ |
| YDW | Wumolong, Yongde county | S.-X. Yang 08B53 | 24°12.9´ | 99°42.6´ |
| YXP | Manwan, Yunxian county | S.-X. Yang 06B99 | 24°37.4´ | 100°19.9´ |
| CNP | Wenquan, Changning county | S.-X. Yang 06F58 | 24°42.8´ | 99°41.0´ |
| FQP | Xiaowan, Fengqing county | S.-X. Yang & D.-W. Zhao 07B02 | 24°36.5´ | 100°4.2´ |
| LLP | Longjiang, Longlin county | S.-X. Yang & Y. Liu 08A54 | 24°44.8´ | 98°44.9´ |
| DLP | Xiaguan, Dali city | S.-X. Yang & Y. Liu 08B05 | 25°36.6´ | 100°10.8´ |
| OJD | Yangjie, Yuanjiang county | S.-X. Yang & Y. Liu 07B85 | 23°26.2´ | 102°3.4´ |
| LXD | Jiangdo, Luxi county | S.-X. Yang & Y. Liu 08A05 | 24°29.7´ | 98°21.3´ |
| YJD | Xima, Yingjiang county | S.-X. Yang & Y. Liu 08A06 | 24°42.9´ | 97°44.3´ |
| ZKD | Zhenkang county | S.-X. Yang 08B52 | 23°50.6´ | 98°58.4´ |

Population LXW is located in Myanmar and the other populations are located in Yunnan province of China.
